# Regulation of ascorbic acid metabolism in postharvest navel orange fruit during storage by exogenous hydrogen sulfide

**DOI:** 10.3389/fnut.2026.1760332

**Published:** 2026-04-02

**Authors:** Danni Lv, Tenghuan Huang, Yule Li, Xiaoxia Zuo, Jing Wang, Zhipeng Cai, Yonggen Shen, Huayign Du, Wei Zhang, Zhenglu Liu, Liqin Zhu

**Affiliations:** 1 College of Food Science and Engineering, Jiangxi Agricultural University, Nanchang, Jiangxi, China; 2 College of Agriculture and Food Engineering, Baise University, Baise, China

**Keywords:** ascorbic acid, gene expression, hydrogen sulfide, metabolic regulation, navel orange, nutritional value

## Abstract

**Background:**

Hydrogen sulfide (H_2_S) is an important gaseous signaling molecule in plants. It has been shown in our previous studies to maintain the storage quality of postharvest fruits at low concentrations. Navel orange, a fruit relatively high in vitamin C (ascorbic acid), experiences a rapid decline in this nutrient during postharvest storage, which reduces its nutritional value. However, the effect of H_2_S on ascorbic acid (AsA) metabolism of postharvest navel orange from molecular level has not been reported. This study investigates the application of exogenous H_2_S to regulate AsA metabolism in navel orange.

**Methods:**

Postharvest navel orange fruits were fumigated with 25 μl L^−1^ H_2_S (air as control) for 30 min, and stored at 20 °C for 18 days. Fruit samples were collected every three days for analysis. The content of total AsA, and AsA was quantified. Concurrently, general quality parameters including weight loss, soluble solids content (SSC), and titratable acidity (TA) were also monitored. The expression levels of key genes involved in AsA metabolic pathway were analyzed using quantitative real-time PCR.

**Results:**

Fumigation with 25 μl L^−1^ H_2_S significantly inhibited the increase in weight loss, maintained SSC, and delayed the decline in TA, total AsA, and AsA content in navel oranges during the 18-day storage period. The expression level of genes involved in the ASA metabolic pathway was modulated by H_2_S treatment. Up-regulated genes of AsA biosynthesis (*CitPGI, CitPMM, CitGME, CitGGP, CitGPP, CitGalDH, CitGalLDH*, and *CitGalUR*) and regeneration (*CitMDHAR* and *CitGR*), as well as down-regulated genes of degradation (*CitAO*), all contributed to the increased AsA content during storage.

**Conclusion:**

Application of exogenous 25 μl L^−1^ H_2_S effectively preserved the postharvest quality and nutritional value of navel orange. Further studies showed that H_2_S primarily regulated the expression of genes involved in the AsA metabolism pathway in fruits via the L-galactose pathway, while cooperating with D-galacturonic acid, AsA cycle and degradation pathways to accelerate the synthesis and accumulation of AsA in fruits. These findings demonstrate that H_2_S treatment helps maintain higher vitamin C content in navel orange by regulating key genes in the AsA metabolic pathway.

## 1 Introduction

Navel orange (*Citrus sinensis*), as a nutrient-dense food widely consumed by humans, is recognized as a primary dietary source of ascorbic acid (AsA, commonly known as vitamin C), which is essential for human nutrition and health (1). It is characterized by its high juice content and suitability for fresh consumption (2). Vitamin C is an indispensable antioxidant and enzymatic cofactor for human health, crucial for immune function and iron absorption (3). It has good protective and therapeutic effects on the human body (4). The bioavailability of vitamin C in oranges has been reported to be as high as 53% (5). However, epidemiological data indicate a widespread prevalence of inadequate intake or subclinical deficiency of vitamin C in modern industrial societies (6). Nevertheless, the content of AsA significantly decreases in orange during postharvest storage, which compromises its nutritional value (7). This loss directly diminishes the anticipated health benefits for consumers.

Hydrogen sulfide (H_2_S) is recognized as a pivotal gaseous signaling molecule that also functions as a fundamental biological catalyst and structural cellular component involved in numerous physiological processes (8). Exogenous inducers such as nitric oxide (NO) and melatonin have been reported to effectively maintain AsA content in fruit during postharvest storage (9, 10). Similarly, evidence shows that H_2_S can also maintain high levels of nutrient-related metabolites, including AsA, thereby preserving better nutritional quality in fruits (11, 12). For instance, previous surveys have reported that H_2_S treatment can help preserve the AsA content of strawberry throughout storage (13). Similarly, H_2_S treatment significantly enhanced AsA accumulation in hawthorn fruit during cold storage, which contributed to improved antioxidant capacity (14). Recent research discovered that H_2_S treatment significantly inhibited the decline of AsA content in fresh-cut apple during storage by enhancing its recycling pathway (15). Meanwhile, Niazi et al. (16) reported that H_2_S treatment maintained a higher AsA accumulation in persimmon fruit during cold storage, thereby improving the nutritional quality of the fruit. At low concentrations, H_2_S acts as a physiological trigger that upregulates the fruit's own defense and maintenance systems. However, the effect of H_2_S treatment of navel orange fruit on AsA accumulation has not been reported.

The regulation of AsA homeostasis in postharvest fruits, involving biosynthesis, recycling, and degradation pathways, likely contributes to the maintenance of its content (17, 18). In the biosynthesis pathway, the L-galactose pathway has been established by Wheeler et al. (19) as the dominant route for AsA synthesis in higher plants. This pathway integrates AsA biosynthesis into central carbohydrate metabolism and connects polysaccharide synthesis with protein glycosylation. Furthermore, L-galactose serves as a direct precursor, and key regulatory enzymes in this route, like GDP-D-mannose-3′,5′-epimerase (GME), GDP-L-galactose phosphorylase (GGP), and L-galactono-1,4-lactone dehydrogenase (GalLDH), have been functionally characterized in species such as tomato and black currant (20–22). In addition, the D-galacturonic acid pathway contributes to AsA accumulation in fruits. In kiwifruit, the expression of galacturonic acid reductase (GalUR) and myo-inositol oxygenase (MIOX) is upregulated during ripening and correlates positively with AsA content (23, 24).

In the recycling pathway, AsA is oxidized to monodehydroascorbate (MDHA) by ascorbate peroxidase (APX) using H_2_O_2_, or by ascorbate oxidase (AO) using O_2_. MDHA can be reduced back to AsA by monodehydroascorbate reductase (MDHAR). Alternatively, AsA may be non-enzymatically converted to dehydroascorbate (DHA), which is then recycled to AsA by dehydroascorbate reductase (DHAR) using glutathione (GSH) as an electron donor. The resulting oxidized glutathione (GSSG) is regenerated to GSH by glutathione reductase (GR), thereby sustaining the AsA–GSH cycle. GR plays a key role in maintaining a high GSH/GSSG ratio, which supports efficient AsA regeneration (25). Additionally, AsA content are influenced by exogenous factors, such as light (26), temperature (27), and hormones (9). For instance, blue LED light upregulates genes involved in AsA biosynthesis and recycling, leading to elevated AsA content in citrus juice sacs (28). Similarly, low temperature (10 °C) enhances AsA accumulation in citrus fruit through modulation of related biosynthetic and recycling genes (29). Furthermore, 1-methylcyclopropene (1-MCP) has been shown to delay the fibrosis process in *Rosa sterilis* fruit, thereby helping to retain AsA content during storage (30). These findings underscore that targeted modulation of AsA metabolism is important for nutrition quality. Therefore, applying novel preservation technologies capable of regulating AsA metabolism is crucial for minimizing postharvest losses and maintaining the nutritional value of navel oranges.

Nevertheless, no research exists on the molecular level regulation of ASA metabolism by H_2_S in postharvest navel orange. Therefore, to investigate whether H_2_S treatment preserves AsA content in postharvest navel oranges during storage, we determined the changes in AsA concentration in the pulp of navel orange fruit during storage and conducted a systematic analysis of the gene expression features associated with its metabolic pathway. The findings are expected to provide novel insights into the physiological role of H_2_S in AsA retention and lay a theoretical foundation for developing H_2_S-based, nutrition-preserving technologies for the citrus industry.

## 2 Materials and methods

### 2.1 Material treatment methods

The navel orange (*Citrus sinensis* cv. Newhall) fruit was harvested from an orchard located in Shanggao County, Nanchang City, Jiangxi province, China. After 24–48 h of heat dissipation at room temperature to dislodge field heat, uniformly sized and flat-surfaced fruit were randomly assigned to two groups for subsequent experiments. Twenty five microliter per liter H_2_S gas (99.99% purity) was selected as the optimum concentration for the experiment, and fruit were placed in a 30 L airtight container and fumigated at 20 °C for 30 min. Meanwhile, fruits were treated with air at 20 °C for 30 min as the control (CK). For each treatment, fruits were divided into two groups: one for weight loss monitoring (*n* = 30), and another for destructive sampling and indicator analysis (*n* = 63). The experiment included three biological replicates, with a total of 186 fruits. Following treatment, each fruit was placed in polyethylene bags (150 mm long × 150 mm wide × 0.01 mm thick), and stored at controlled environmental laboratory (20 ± 0.5 °C, 85–90% relative humidity) for 18 days. At each sampling time point (days 0, 3, 6, 9, 12, 15, and 18), nine fruits from each treatment group were collected, immediately frozen in liquid nitrogen, and stored at −80 °C.

### 2.2 Measurement of weight loss

The fresh weight loss in 30 fruits per treatment was determined after 3, 6, 9, 12, 15 and 18 d of storage. The following formula was used to determine weight loss:


$$\begin{array}{l} \text{Weight loss }\left(\text{\%}\right)=\frac{\text{Initial fruit weight}-\text{Weighing weight}}{\text{Initial fruit weight }}\times 100 \end{array}$$

### 2.3 Measurement of SSC and TA content

The SSC of every pulp sampled every three days was determined using a portable digital refractometer (ATAGO PAL-1, Japan) and expressed as %, and repeated three times.

The TA content was determined using the Acid-base neutralization titration method, described by Zhu et al. (31). Approximately 10.0 g navel orange pulp tissue was ground and homogenized in a mortar, and then transferred into a 100 ml volumetric flask, diluted with distilled water to achieve the volumetric flask's capacity, extracted for 10 min, and filtered. After adsorbing 5.0 ml filtrate into a triangular flask, 2–3 drops of 1% phenolphthalein indicator solution were added, and titrated with 0.5 M NaOH calibration solution until the solution was pink and did not fade within 30 s, which was the titration endpoint. The experiment was repeated three times with distilled water used as a blank control.

### 2.4 Measurement of total AsA and AsA content

The total AsA and AsA contents were measured spectrophotometrically, as depicted by Zhang et al. (32) 10.0 g navel orange pulp samples were weighed, ground it in liquid nitrogen, 20 ml 50 g L^−1^ trichloroacetic acid (TCA) was added to extract homogenate, and then centrifuged at 4 °C (12,000 g, 25 min) to collect the supernatant for use in determining AsA concentration. Meanwhile, 0.5 ml 60 mm dithiotreitol-ethanol was added to the remaining 1.0 ml of the aforementioned supernatant, and the pH was calibrated to seven to eight with 0.2 M Na_2_HPO_4_-1.2 M NaOH and placed at room temperature for 10 min to reduce DHA. Following that, 0.5 ml 0.0002 g L^−1^ TCA was added to calibrate the pH to 1–2 for the purpose of measuring total AsA content.

The 5.0 ml reaction system utilized to assess the total AsA and AsA contents consisted of 1.0 ml supernatant, 1.0 ml 50 g L^−1^ TCA, 1.0 ml absolute-ethanol, 0.5 ml 0.4% phosphoric acid-ethanol, 1.0 ml 5 g L^−1^ 4, 7-diphenyl-1, 10-phenanthroline-ethanol, and 0.5 ml 0.3 g L^−1^ ferric chloride-ethanol, which was incubated at 30 °C for 60 min and the absorbance measured at 534 nm, and repeated three times.

### 2.5 Total RNA extraction and RT-qPCR analysis

Total RNA was extracted from each navel orange sample using the plant RNA extraction kit (Huayueyang, Beijing, China). The cDNA was synthesized by reverse transcription using the HiScript III-RT reverse transcription kit (Vazyme, Nanjing, China) and the extracted total RNA as a template. The optimized ASA anabolic gene sequences for navel orange were derived mostly from the sweet orange genome database (http://citrus.hzau.edu.cn/index.php) (33). The primer sequences are listed in Supplementary Table S1. RT-qPCR analysis was performed on a Bio-Rad CFX96 system using ChamQ Universal SYBR qPCR Master Mix (Vazyme, China). Three biological repetitions were performed at each sample time point and handling group. The transcription levels of associated genes were normalized to *CitActin* and computed using the 2^−Δ*ΔCt*^ method.

### 2.6 Statistical analysis

Data were analyzed using SPSS 25.0 and presented as the mean ± standard error (SE). One-way ANOVA analysis and Duncan's multiple range tests were used to examine the difference between data, and *P* < 0.05 denoted statistical significance. Graphs were generated with Origin 2018.

## 3 Results

### 3.1 Weight loss, SSC, and TA content

**Figure 1:**
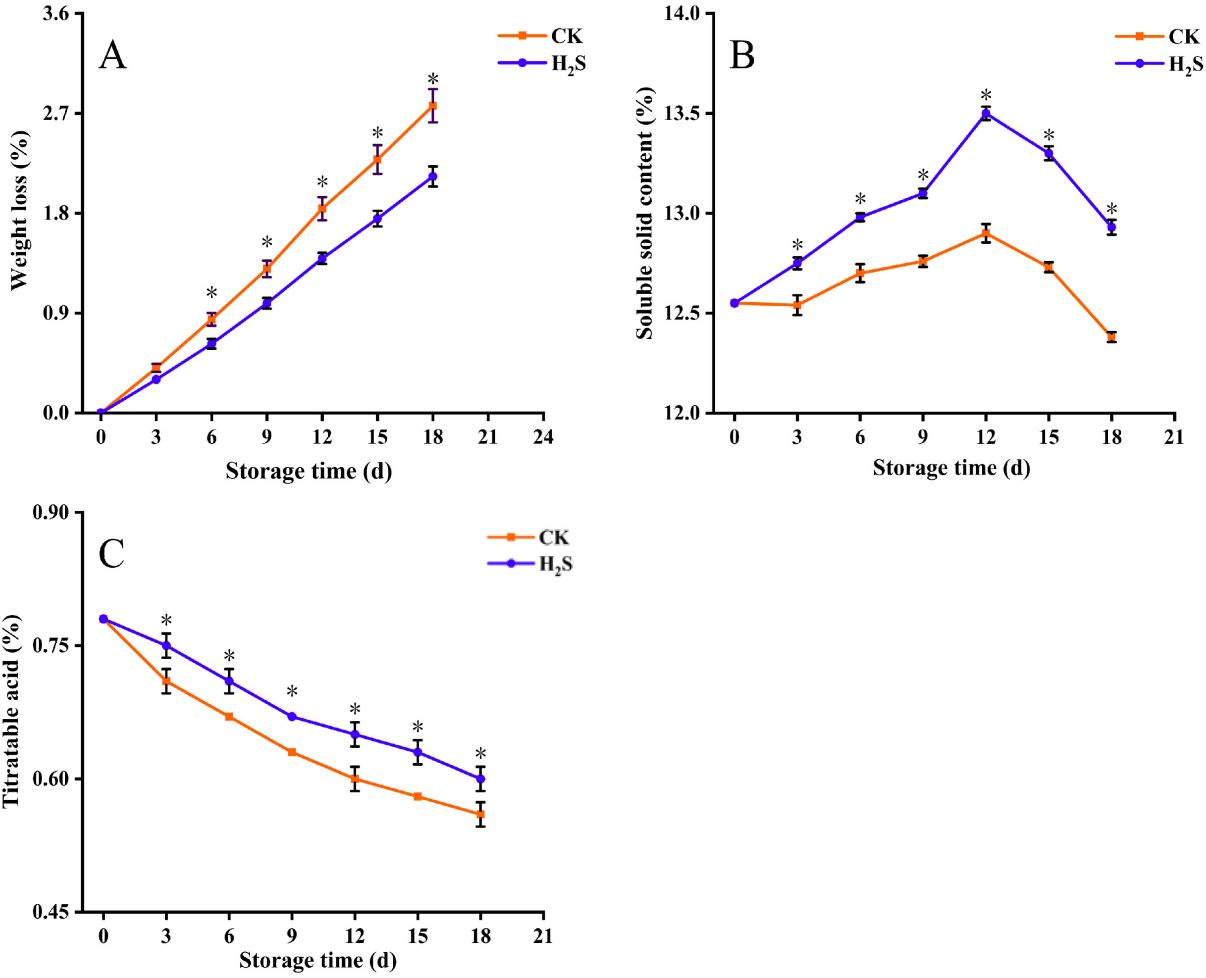
Weight loss **(A)**, SSC **(B)**, and TA content **(C)** of navel orange fruit after H_2_S treatment during storage at 20 °C. Vertical bars indicate SE. Asterisks show the significant differences between CK and H_2_S treatment (*P* < 0.05).

During the 18-day storage period, weight loss increased with storage time in both H_2_S-treated and CK groups. The fruit weight loss of H_2_S treatment was significantly lower than that of CK (*P* < 0.05; Figure 1A). SSC increased at first and then decreased as storage time increased during the 18-day storage period. The SSC of H_2_S-treated group was significantly higher than that of CK throughout the storage period (*P* < 0.05; Figure 1B). Although the TA content declined gradually in all samples during storage, the H_2_S-treated group maintained a consistently and significantly higher TA level than the CK throughout the 18-day storage period (*P* < 0.05; Figure 1C).

### 3.2 Total AsA, and AsA content

**Figure 2:**
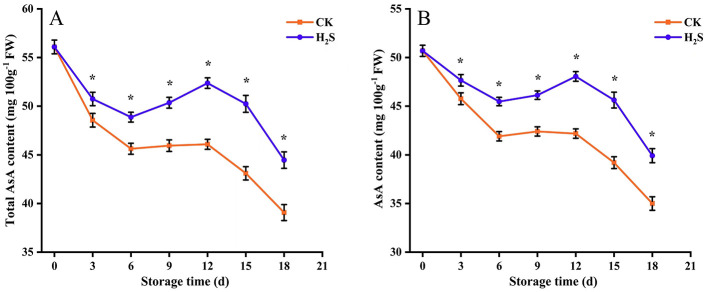
Total AsA content **(A)**, and AsA content **(B)** of navel orange fruit after H_2_S treatment during storage at 20 °C. Vertical bars indicate SE. Asterisks show the significant differences between CK and H_2_S treatment (*P* < 0.05).

The total AsA content showed a similar trajectory to that of AsA, increasing slowly during the intermediate storage period and decreasing during the latter storage period (Figure 2). The total AsA and AsA contents in the H_2_S treatment group were significantly higher than those in the CK throughout the 18-day storage period (*P* < 0.05). Notably, the total AsA and AsA contents of H_2_S-treated fruits reached the highest level, and both of them were 1.14-fold that of CK on day 12.

### 3.3 Expression of AsA synthesis-related genes in navel orange fruit after H_2_S treatment

**Figure 3:**
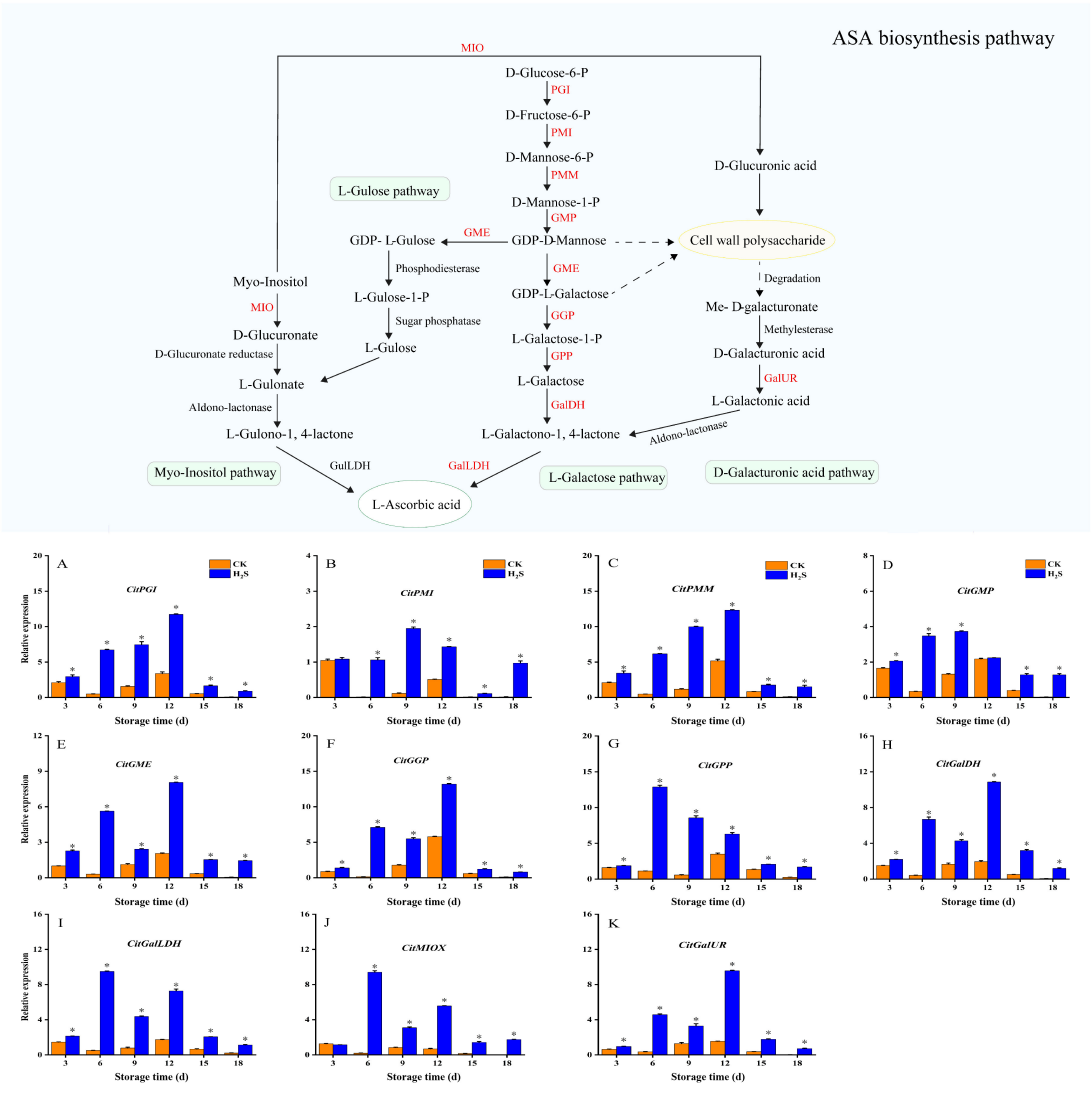
Expression levels of CitPGI **(A)**, CitPMI **(B)**, CitPMM **(C)**, CitGMP **(D)**, CitGME **(E)**, CitGGP **(F)**, CitGPP **(G)**, CitGalDH **(H)**, CitGalLDH **(I)**, CitMIOX **(J)** and CitGalUR **(K)** in navel orange fruit treated with H_2_S during storage at 20 °C. Vertical bars indicate SE. Asterisks show the significant differences between CK and H_2_S -treated (*P* < 0.05).

The expression levels of *CitPGI* and *CitPMM* increased (Figures 3A, C), whereas *CitGPP* decreased during 18-day storage (Figure 3G). Besides, the expression levels of other AsA biosynthetic genes exhibited an upward to a downward trend. The expression levels of *CitPGI, CitPMM, CitGME, CitGGP, CitGPP, CitGalDH, CitGalLDH* and *CitGalUR* in H_2_S-treated group were dramatically higher than those in CK during the 18-day storage period (*P* < 0.05; Figures 3A, C, E–I, K). Meanwhile, the expression levels of *CitPMI* and *CitMIOX* in H_2_S-treated group were significantly higher than in CK on days 6, 9, 12, 15, 18 (*P* < 0.05; Figures 3B, J). And the expression level of *CitGMP* in H_2_S-treated group was markedly higher than in CK on days 3, 6, 9, 15, 18 (*P* < 0.05; Figure 3D).

### 3.4 Expression of AsA cycle and degradation-related genes in navel orange fruit after H_2_S treatment

**Figure 4:**
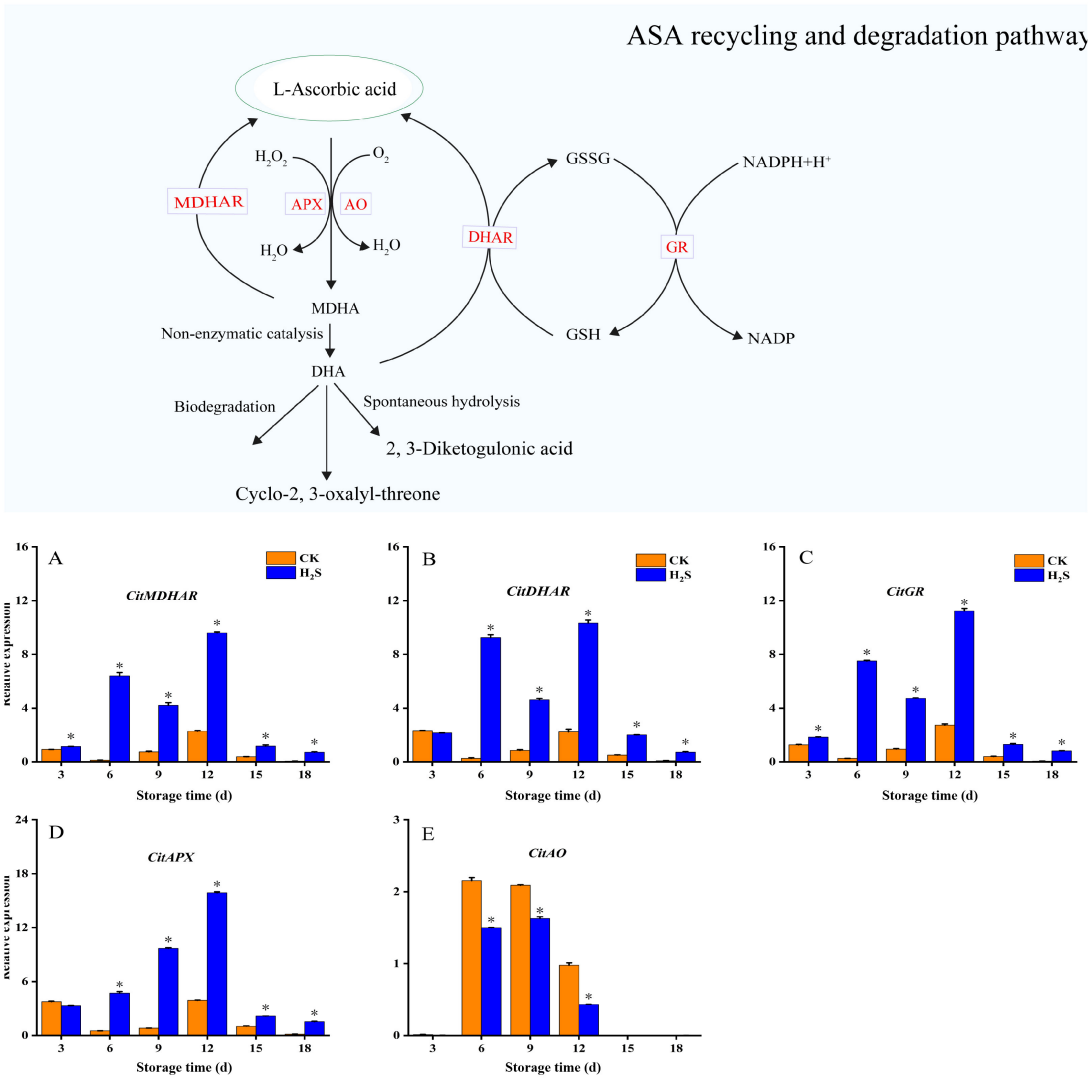
Expression levels of CitMDHAR **(A)**, CitDHAR **(B)**, CitGR **(C)**, CitAPX **(D)** and CitAO **(E)** in navel orange fruit treated with H_2_S during storage at 20 °C. Vertical bars indicate SE. Asterisks show the significant differences between CK and H_2_S-treated (*P* < 0.05).

The expression levels of *CitMDHAR* and *CitGR* in H_2_S-treated group were significantly higher than those in CK during the 18-day storage period (*P* < 0.05; Figures 4A, C). Compared with CK, H_2_S treatment significantly increased the expression levels of *CitDHAR* and *CitAPX* throughout the storage period, except on day 3 (*P* < 0.05; Figures 4B, D). Nonetheless, the expression level of *CitAO* in H_2_S-treated group were significantly lower than those in CK (Figure 4E).

## 4 Discussion

The effects of H_2_S on fruit physiology have become increasingly well understood in recent years (34). This study found that H_2_S treatment significantly inhibited the rate of weight loss, while simultaneously maintaining SSC and delaying the decrease of TA content in navel orange fruits, which is consistent with previous reports that H_2_S maintains SSC and delays the decline of TA and AsA in litchi (35). More importantly, H_2_S treatment significantly delayed the decrease of total AsA and AsA content in navel orange. Previous studies have demonstrated that H_2_S treatment maintained higher AsA levels in citrus and grape (36, 37). Therefore, H_2_S fumigation could directly help to maintain the nutrition of navel orange. Consumers purchasing navel orange treated with H_2_S would ingest a greater quantity of the vital nutrient (vitamin C) compared to fruits subjected to conventional storage. Vitamin C performs vital functions such as collagen synthesis, antioxidant activity, and immune support, it also enhances the absorption of non-heme iron, calcium, and magnesium, and synergizes with vitamin E (38, 39).

In this study, H_2_S treatment significantly increased the expression levels of key genes involved L-galactose pathway (*CitPGI, CitPMI, CitPMM, CitGMP, CitGME, CitGGP, CitGPP, CitGalDH, CitGalLDH*), the myo-inositol pathway (*CitMIOX*) and the D-galacturonic acid pathway (*CitGalUR*). Studies have revealed that AsA content may be affected by genetic factors, thus the majority of the genes participated in the L-galactose pathway are the key regulatory genes for AsA accumulation (40–42). For example, 1-MCP was used to promote the expression of key AsA accumulation genes (*AdGME, AdGalDH*, and *AdGalLDH*) in kiwifruit (32). Alos et al. (43) also found that transcripts from L-galactose pathway genes including *GGP, GPP, MDHAR3*, and *DHAR1* had positive effects on AsA accumulation in citrus fruit. Therefore, the increase in AsA content during early fruit storage is most likely related to the high transcription abundance of L-galactose pathway genes. The results showed that H_2_S treatment might promote AsA accumulation throughout fruit storage by inducing the expression levels of L-galactose pathway key genes. Meanwhile, previous studies have found that the level of *GalUR* gene transcription has a positive effect on AsA accumulation in tomato (21), and strawberry (44). In addition, overexpression of *MIOX4* resulted in increased light response and AsA biosynthesis ability (45). Therefore, the myo-inositol pathway may be involved in AsA synthesis in the initial stage of fruit development, and then mainly involved in L-galactose and D-galacturonic acid pathway in the final stage of cell enlargement and the whole fruit ripening period. Caruso et al. (46) recently discovered that the change in AsA content in sweet orange juice was also associated with a decrease in *GalUr12* and *MyoOx* transcription. Thus, The findings indicated that D-galacturonic acid and myo-inositol pathways contributed to the up-regulation of AsA-biosynthesis gene level in navel orange fruit by H_2_S-treated, and D-galacturonic acid pathway seems to play a more important role than myo-inositol pathway in promoting AsA accumulation in navel orange fruit by H_2_S, but the specific mechanism needs further study.

Apart from biosynthesis, it has been extensively documented that the AsA cycle and degradation promote AsA accumulation in the AsA-GSH cycle (42, 47). Therefore, we examined the coding genes (*CitMDHAR, CitDHAR, CitGR*) for AsA-regeneration to gain a better comprehension of the modulation of H_2_S treatment on AsA metabolism in navel orange fruit. There have been studies that overexpression of *DHAR* increased AsA level in tomato (48). The up-regulation expression of the *CitMDHAR, CitDHAR*, and *CitGR* genes in navel orange fruit treated with H_2_S could enhance the accumulation of AsA. Apart from that, we investigated two AsA degradation genes, *CitAPX* and *CitAO*. As a major scavenging enzyme in the body's oxidation system, APX contributes significantly to H_2_O_2_ scavenging (49). Furthermore, overexpression of the *MaAPX* gene improved banana fruit cold tolerance (50). In our investigation, fumigating navel orange fruit with exogenous H_2_S induced the up-regulation of *CitAPX* gene expression during storage, which may promote the process of reducing MDHA to AsA by MDHAR to accelerate AsA accumulation in the fruit, and further promote APX activity, accelerate H_2_O_2_ clearance to improve fruit storability. The results established that H_2_S significantly reduced the expression level of *CitAO*. Sanmartin et al. (51) found that AO transcripts were higher in the early stage of fruit development, because AO was involved in the cell growth of citrus fruits (division and expansion). Hence, down-regulation of *CitAO* transcription may result in decreased AO activity, inhibiting AsA oxidation while simultaneously activating the changes of some biosynthesis and recycling genes, and promoting the source of higher AsA content in fruit to meet the requirement for AsA under different growth conditions. This was consistent with the findings in a study of melons (52). The findings showed that both APX and AO enzymes potentially contribute to AsA degradation and are required to regenerate AsA via MDHA production. Thus, H_2_S treatment showed a strong correlation with AsA cycling and degradation in navel orange fruit, particularly *CitMDHAR, CitGR*, and *CitAO*, which may play a critical role in regulating AsA content in navel orange.

Future application of H_2_S treatments in the supply chain could help reduce postharvest nutrient losses and deliver healthier fresh produce to the market. The H_2_S fumigation treatment in this study could be readily adapted as a final step before the post-harvest refrigeration or packaging of navel orange. The primary regulatory hurdle will be the safety assessment and establishment of residue limits for H_2_S on treated fruit. While the effect of H_2_S in preserving nutritional value is clear, its practical application necessitates consideration of consumer perception and acceptance. H_2_S is a volatile, endogenous plant metabolite with signaling functions, detectable in tissue emissions particularly from Allium species, and involved in physiological processes and stress responses (53). For example, garlic releases trace amounts of hydrogen sulfide, which acts as a beneficial signaling molecule in the body, exerting protective effects without causing harm at physiological concentrations (54). It is worth highlighting that the H_2_S treatment employs biologically relevant, minuscule amounts (25 μL L^−1^) solely as a signal to the fruit, without imparting any residual odor. Importantly, this signal results in a tangible consumer benefit: navel orange that retains more of its natural vitamin C.

## 5 Conclusion

In conclusion, application of 25 μL L^−1^ H_2_S effectively maintained the AsA content, while reducing weight loss and delaying the decrease of SSC and TA content in navel orange fruit during the 18-day storage period. At the molecular level, H_2_S primarily up-regulated the expression levels of genes involved in the ASA metabolism pathway in fruit by the L-galactose pathway (*CitPGI, CitPMI, CitPMM, CitGMP, CitGME, CitGGP, CitGPP, CitGalDH, CitGalLDH*), and up-regulated genes involved in the synergistic pathway (*CitMIOX, CitGalUR*) and regeneration pathway (*CitMDHAR, CitMDHAR, CitGR*), as well as down-regulated genes involved in the degradation pathway (*CitAO*). This study demonstrates that H_2_S fumigation maintains AsA content in postharvest navel orange fruit by modulating the expression of genes involved in AsA metabolism.

## Data Availability

The raw data supporting the conclusions of this article will be made available by corresponding authors upon reasonable request.
