# Supplementary material for: Regulation of ascorbic acid metabolism in postharvest navel orange fruit during storage by exogenous hydrogen sulfide
Source: Front Nutr. 2026 Apr 2;13:1760332. doi: 10.3389/fnut.2026.1760332 (PMC13083027; doi:10.3389/fnut.2026.1760332)
Supplement: Supplementary file 1 [file Table_1.docx]

**Supplementary Table S1.** Sequences of primers used in quantitative real-time RT-qPCR. The analysed genes are *PGI, glucose-6-phosphate isomerase; PMI, phosphomannose isomerase; PMM, phosphomannomutase; GMP, GDP-mannose pyrophosphorylase; GME, GDP-_D_-mannose-3’, 5’-epimerase; GGP, GDP-_L_-galactose phosphorylase; GPP, _L_-galactose-1-phosphate phosphatase; GalDH, _L_-galactose dehydrogenase; GalLDH, _L_-galactono-1, 4-lactone dehydrogenase; MIOX, myo-inositol oxygenase; GalUR, galacturonic acid reductase; MDHAR, monodehydroascorbate reductase; DHAR, dehydroascorbate reductase; GR, glutathione reductase; AO, ascorbate oxidase; APX, ascorbate peroxidase*.

| Gene name | Accession number | Primer sequence (5’-3’) |
| --- | --- | --- |
| PGI | Cs3g24700.1 | F: TCTGCAGTTGCAAAGCACAT |
|  |  | R: CGACAGCACTGCAAACACTA |
| PMI | Cs2g12720.1 | F: CAGTAGCCGGACCATCTCTT |
|  |  | R: CATGTAGGCAGGCACAAACA |
| PMM | orange1.1t00331.1 | F: CCAGGGATGGGACAAGACAT |
|  |  | R: GCCAGGAAGAGAGCCTTACA |
| GMP | Cs7g31960.1 | F: TTGCTAACAAACCCATGATCC |
|  |  | R: CAACATCACCTCTGGCTGGT |
| GME | Cs3g10840.1 | F: GGAGAAGCTTGCAAGTGAGG |
|  |  | R: AGCAGGAGCTTTTTCCCTTC |
| GGP | Cs4g09870.2 | F: CAGGGTCATTCCTGGTCAGT |
|  |  | R: TGGACTTCACCATCTTCGCT |
| GPP | Cs2g02090.1 | F: TCTTGGACACTGCAGTTGATG |
|  |  | R: TCTGTGACCAAATCCACCTG |
| GalDH | Cs8g03180.1 | F: GGCGTTCCGAGAAATGAGTA |
|  |  | R: CTCGCCAAGCTCTCATCAAT |
| GalLDH | orange1.1t00763.1 | F: ATCTCCTGACGGCACTGAAC |
|  |  | R: TCCCTCTGACTTCCTCCAGA |
| MIOX | Cs1g16030.1 | F: TGCCACAGACCAACTCATTT |
|  |  | R: TGCCATACTCCTCCCTCATC |
| GalUR | Cs7g07970.1 | F: CCAGGTGTGTTTAAGATGGGCATACG |
|  |  | R: CTTTGTGGGATATCACTGATTTTCTTGGTC |
| MDHAR | Cs5g03080.1 | F: GAAGCTGGTTCTGACGGACGT |
|  |  | R: CTGACCATCAACCTGTATTCCACCCAC |
| DHAR | Cs6g17520.1 | F: CCCAGATCCGCCACTACGAACC |
|  |  | R: CCCCAATGATAAAAGGGCCATTTTC |
| GR | orange1.1t03206.3 | F: GAGCCAGCCCAATCAAGAAG |
|  |  | R: CCACGAATGACACAGGTTCC |
| APX | Cs6g04140.2 | F: TGGAAGACCGGACAAATCTGATCCG |
|  |  | R: TTATCACTCAGACCCATGTGGCCG |
| AO | Cs2g29090.1 | F: CGGGTTCACTAAGTGGGCTA |
|  |  | R: TGGAGGTTTCATCACGTCATAC |
| Actin | Cs1g05000 | F: TGGATTCTGGTGATGGTGTG |
|  |  | R: GTTCGGCTGTGGTGGTAAAC |

Note: Target genes were identified from the Huazhong Agricultural University sweet orange genome database (http://citrus.hzau.edu.cn/index.php).
